# Peripheral lncRNA–IL1RAP Dysregulation in Schizophrenia: A Multi‐Omics Bridge Between Immunity and Diagnosis

**DOI:** 10.1002/brb3.71384

**Published:** 2026-04-16

**Authors:** Jie Wu, Ruize Niu, Zijun Liu, Yuanyuan Li, Yunqiao Zhang, Zhaowei Teng, Yong Zeng, Tianhao Bao

**Affiliations:** ^1^ The Affiliated Mental Health Center Kunming Medical University Kunming Yunnan China; ^2^ The Sixth Affiliated Hospital, Kunming Medical University Yuxi Yunnan China; ^3^ The Second Affiliated Hospital of Kunming Medical University, Yunnan Key Laboratory of Neuropsychiatric Disorders Kunming Yunnan China

**Keywords:** IL‐1/IL1RAP signaling, integrative transcriptomics, long non‐coding RNA, peripheral immune dysregulation, schizophrenia

## Abstract

**Background:**

Schizophrenia (SCZ) is frequently accompanied by peripheral immune dysregulation, yet robust and reproducible blood‐based molecular markers remain limited.

**Objective:**

We sought a compact long non‐coding RNA (lncRNA) signal in peripheral blood leukocytes (PBL) and examined how it aligns with immune‐linked transcriptional programs and cellular compartments, using bulk RNA sequencing (RNA‐seq), targeted validation, and a healthy‐donor peripheral blood mononuclear cell (PBMC) single‐cell reference for localization.

**Methods:**

PBL from 50 first‐episode or unmedicated SCZ patients and 50 matched controls underwent RNA‐seq. We performed reference‐guided transcript annotation and generated lncRNA and mRNA count matrices. Differential expression was assessed with edgeR (TMM normalization). Candidate lncRNAs were prioritized using sample‐level lncRNA‐mRNA co‐variation, followed by qRT–PCR validation in an independent cohort and evaluation with a two‐feature logistic regression model using repeated 10‐fold cross‐validation. Pathway‐scale analyses and weighted gene co‐expression network analysis (WGCNA) summarized coordinated programs. Single‐cell data from healthy donors were used for expression localization only, not case‐control testing.

**Results:**

We observed a small set of dysregulated lncRNAs alongside broader mRNA changes. Two candidates at the CCR3 and IL1RAP loci (TCONS_00134168/lncRNA–CCR3 and TCONS_00138311/lncRNA–IL1RAP) showed consistent case–control directionality and were supported by qRT–PCR. The two‐lncRNA model showed strong internal discrimination (AUC = 0.933) but weaker, uncertain performance in a small external qRT–PCR set (AUC = 0.656). Enrichment analyses highlighted synapse‐related annotations, RNA processing/translation, and immune signaling, with recurrent involvement of IL1RAP‐linked IL‐1 branches. WGCNA placed lncRNA–IL1RAP and IL1RAP within diagnosis‐inversely associated co‐expression programs, whereas lncRNA–CCR3 showed a more transcript‐specific pattern. In the healthy‐reference single‐cell atlas, IL1B/IL1RAP/HSF1 signals were most prominent in the monocyte/macrophage compartment.

**Conclusion:**

Together, these findings support an exploratory two‐lncRNA candidate marker concept, while underscoring the need for larger multi‐center validation and targeted mechanistic follow‐up without implying causality.

## Graphical abstract

Bulk RNA‐seq of PBL from first‐episode or unmedicated SCZ patients identifies an opposite‐direction two‐lncRNA signal at the CCR3 and IL1RAP loci, supported by qRT–PCR and yielding strong internal but uncertain external discrimination. Immune‐linked programs recurrently involve IL1RAP/IL‐1 branches and map, in a healthy‐donor PBMC single‐cell reference, to a monocyte/macrophage compartment enriched for IL1B/IL1RAP/HSF1.

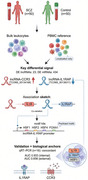

## 1 Introduction

Schizophrenia (SCZ) is a severe mental disorder with an unknown etiology and a high lifetime prevalence, contributing substantially to the global health burden (Velligan and Rao 2023; Striebel 2024; Gupta et al. 2015; Solmi et al. 2023). In routine practice, SCZ is still diagnosed mainly through symptom‐based assessments, which remain subjective and can be difficult to standardize (Kendler 2016). Etiologic studies increasingly point to a complex, polygenic architecture (Sullivan et al. 2024; Ruzicka et al. 2024; Ling et al. 2024; Fröhlich et al. 2024), and a growing body of work also implicates dysregulation of the peripheral immune system in SCZ pathology (Pan et al. 2025; Zhu et al. 2023).

In this context, long non‐coding RNAs (lncRNAs) in peripheral blood have drawn attention because they show measurable dysregulation in SCZ and are linked to immune and inflammatory regulatory pathways (Jia et al. 2025; Tian et al. 2018; Ren et al. 2020). At the molecular level, lncRNAs can influence processes relevant to disease biology, including neuronal activity, neurotransmitter metabolism, and synaptic plasticity (Mishra and Kumar 2021; Du et al. 2023; Mukhopadhyay et al. 2023). Because some regulatory programs are shared between the brain and peripheral blood, readily accessible blood lncRNAs may provide a practical “window” into central pathological changes (Song et al. 2021; Luykx et al. 2016; Robinson 2014; Cao 2014; Lim et al. 2022; Miyano et al. 2024). A key limitation, however, is that bulk transcriptome sequencing cannot resolve cellular heterogeneity, making it hard to assign signals to specific cellular sources (Yang et al. 2023). Single‐cell RNA sequencing (scRNA‐seq) offers a way to address this gap by profiling complex cell compositions and identifying distinct subpopulations enriched for SCZ risk genes (Ruzicka et al. 2024; Wu et al. 2023; Batiuk et al. 2022).

To obtain a more systematic view of peripheral immune dysregulation in SCZ, we integrated transcriptomic data from patient‐derived peripheral blood leukocytes (PBL) with publicly available single‐cell datasets. We aimed to identify lncRNAs linked to immune regulation in SCZ, evaluate their diagnostic potential, and examine their functional context at single‐cell resolution.

## 2 Methods

### 2.1 Cohort Enrollment and Peripheral Blood Processing

Peripheral blood samples for RNA sequencing (RNA‐seq) were collected from psychiatric inpatients and outpatients at the Sixth Affiliated Hospital of Kunming Medical University, the Second People's Hospital of Honghe Prefecture, and the Second People's Hospital of Yuxi City. The SCZ group included 50 individuals diagnosed according to DSM‐5 criteria, who were either at first episode or had not received antipsychotic medication within the 5 weeks prior to enrollment (Tandon et al. 2013). All participants were of Han ancestry and 20–50 years of age. Venous blood (5–10 mL) was drawn into EDTA anticoagulant tubes and processed within 60 min; leukocyte fractions were collected and total RNA was extracted using TRIzol (Ambion). RNA purification and library construction were performed using the VAHTS Total RNA‐seq (H/M/R) Library Prep Kit for Illumina (NR603), and libraries were sequenced on an Illumina HiSeq 4000 platform. Cohort‐level matching of age and sex between SCZ and controls is summarized in Supplemental Material (Figure S1 and Supplementary Table S1), and sequencing/input QC metrics are provided in the Supplementary tables (Supplementary Table S2 and Table S3).

### 2.2 Count‐Based Differential Expression Analysis and Identifier Harmonization

Reads were stringently filtered to high‐quality clean reads, rRNA‐derived reads were removed, and the remaining reads were aligned to GRCh38/hg38 with splice‐aware mapping, followed by reference‐guided transcript assembly and merging. Novel lncRNAs were integrated with known annotations, and gene‐level counts were summarized into separate lncRNA and mRNA count matrices. Differential expression between SCZ and controls was tested on raw counts for lncRNAs and mRNAs in parallel using edgeR (TMM normalization and quasi‐likelihood negative binomial modeling). Lowly expressed features were removed with filterByExpr, and differentially expressed features were defined using prespecified thresholds (FDR < 0.05 and |log2FC| ≥ 1, Benjamini–Hochberg correction). TMM‐normalized logCPM values were used for visualization and downstream analyses. For consistent reporting, Ensembl transcript IDs were mapped to gene symbols using biomaRt; features without valid symbols–particularly assembled transcripts–retained their assembly identifiers (TCONS_) as placeholders to preserve traceability, with the full mapping provided in the Supplementary Materials.

### 2.3 Integration Analysis of Single‐Cell Data From Public Databases

We assembled and harmonized human PBMC scRNA‐seq profiles from 10 datasets (GSE138266, GSE149313, GSE149689, GSE150728, GSE162577, GSE168522, GSE192693, HRA000150, PMID:32114394, and an in‐house healthy single‐cell cohort), each comprising clinically screened control individuals without active neuropsychiatric disorders. Raw reads were processed with Cell Ranger (v7.0.1) to generate gene–cell count matrices. For quality control, we retained cells with >200 detected genes and <10% mitochondrial transcript proportion, and removed putative doublets using DoubletFinder (v2.0.3). To minimize batch effects across studies, datasets were integrated in Seurat (v4.2.2) using reciprocal PCA (RPCA)–based anchor finding (mutual nearest neighbors), with a balanced subset of samples across sex and age strata designated as the reference and remaining samples treated as queries; integrated expression matrices were generated with FindIntegrationAnchors/IntegrateData, followed by graph‐based clustering (FindNeighbors/FindClusters) and visualization by UMAP and t‐SNE. Cell identities were assigned with SingleR by correlating each cell's expression profile to reference atlases, and were further evaluated using established marker genes from the literature; low‐quality or ambiguous clusters lacking coherent marker signatures, exhibiting low transcript complexity, or showing elevated mitochondrial content were excluded. In parallel, immune cell composition was estimated from bulk‐like expression matrices using CIBERSORT with linear support vector regression; only samples meeting CIBERSORT significance (*p* < 0.05) and cell fractions >0 were retained for downstream correlation analyses, which were visualized using pheatmap (correlation heatmaps) and ggpubr (scatterplots). Bulk RNA‐seq was generated from PBL, whereas the single‐cell reference atlas was derived from PBMC; therefore, cell‐type interpretation was restricted to immune compartments shared between the two sources.

### 2.4 Transcriptome‐Wide lncRNA–mRNA Co‐Variation Analysis

To place the two prioritized lncRNAs in a sample‐level context, we quantified their expression co‐variation with transcriptome‐wide mRNAs using TMM‐normalized logCPM profiles across all 100 individuals. For each lncRNA, Pearson correlations were computed across samples, and mRNAs were ranked by |*r*| to nominate the strongest co‐varying partners. For visualization, the top five mRNA associations per lncRNA were displayed, with two‐sided *p* values from correlation tests and Benjamini‐Hochberg FDR control applied across the 10 displayed tests. Scatter plots show the fitted linear trend with a 95% confidence band, and each panel reports *r*, *p*, and FDR.

### 2.5 qRT–PCR Validation, Model Evaluation, and Sequence‐Based Annotation

Independent experimental validation was performed by qRT–PCR using β‐actin as the internal control. Relative expression was calculated using the 2^−ΔΔCt method. For model input and ROC‐based evaluation, ΔCt values were computed relative to β‐actin and −ΔCt was used as an expression proxy so that higher values reflect higher abundance. Group differences in qRT–PCR measurements between SCZ and controls were assessed using two‐sided independent‐samples *t*‐tests (*p* < 0.05). For the two‐lncRNA logistic regression model, effect sizes were reported as odds ratios (ORs) with 95% confidence intervals, and probability calibration was evaluated using quantile‐binned calibration curves referenced to the identity line (*y* = *x*). Statistical analyses were performed in R for model development and validation, with supplementary processing conducted in SPSS v22.0. Primer sequences were: lncRNA–IL1RAP (TCONS_00138311), AGGGGAAGGGAATCAACAAATAG (F) and GGGGCGTGGCATGTAACC (R); lncRNA–CCR3 (TCONS_00134168), TCAAGACTTCGTGGCTTAAACAATA (F) and GGAACTCCATACCTGAAAGACCCTA (R).

To generate hypothesis‐supporting annotations for qRT–PCR‐validated transcripts, we performed sequence‐based scans using PROSITE. When needed, NCBI BLAST was used to assess sequence similarity and potential overlap with known gene transcripts, and genomic context was cross‐referenced using the UCSC Genome Browser. Candidate transcription factor binding motifs were queried using JASPAR. These in silico annotations were used to guide interpretation and prioritization and were not used to infer causality.

### 2.6 Two‐lncRNA Logistic Regression Modeling and Cross‐Validated Evaluation

We constructed a two‐feature logistic regression model using the expression of TCONS_00134168 (lncRNA–CCR3) and TCONS_00138311 (lncRNA–IL1RAP) to estimate the individual probability of SCZ and to benchmark the qRT–PCR findings within a unified predictive framework. For RNA‐seq, predictors were TMM‐normalized logCPM values. Model discrimination was evaluated using repeated 10‐fold cross‐validation, summarized by ROC AUC with 95% confidence intervals, and the distribution of AUC across repeats was used to reflect resampling stability. To avoid information leakage during cross‐validation, z‐score parameters were estimated from each training fold and then applied unchanged to the corresponding held‐out fold.

### 2.7 Gene‐Set Enrichment Anchored on lncRNA‐mRNA Co‐Variation

To connect differential‐expression signals to lncRNA‐associated transcriptional programs, we recalculated lncRNA‐mRNA associations using Spearman correlations across all samples using TMM‐normalized logCPM values, restricting the analysis to dysregulated lncRNAs and mRNAs identified in the edgeR SCZ‐versus‐control comparison. Correlated mRNAs passing the prespecified cutoff were retained as the candidate gene set; when the resulting set was too small to support stable enrichment inference under the primary criterion (FDR < 0.05), the inclusion rule was relaxed to nominal *p* < 0.05, and the final gene lists and thresholds used were recorded in the exported correlation tables and filtering logs. We then performed pre‐ranked GSEA on the candidate mRNAs, ordering genes by a signed differential‐expression statistic (direction from logFC; magnitude from the test statistic). Enrichment was evaluated against offline MSigDB GMT collections (GO Biological Process and KEGG MEDICUS), with significance estimated by permutation and multiple testing controlled by Benjamini–Hochberg FDR.

To summarize redundancy among enriched KEGG gene sets, we computed Jaccard similarity between leading‐edge gene sets and constructed a sparse pathway network by retaining, for each pathway, a fixed number of its strongest similarity links above a minimum threshold. Pathway modules were then derived by hierarchical clustering (*k* = 8), and one representative pathway name was used to label each module; modules containing IL1RAP were annotated with “★”.

### 2.8 Co‐Expression Network Analysis by WGCNA

We applied WGCNA to peripheral blood lncRNA and mRNA expression profiles to identify diagnosis‐linked co‐expression programs. Raw counts were TMM‐normalized and transformed to logCPM, and zero‐variance features were removed. Under the noforce setting, we retained the most variable transcripts for network construction (mRNAs: top 8000 by variability; lncRNAs: features retained under the same filtering scheme). Samples were screened by hierarchical clustering to identify extreme outliers prior to network inference. Signed networks were constructed using a soft‐thresholding power (β) selected to approximate scale‐free topology (target signed *R^2^* = 0.85). Modules were defined from topological overlap‐based clustering with dynamic tree cut (minimum module size = 30) and merged based on eigengene similarity (merge cut height = 0.25). Module eigengenes were correlated with SCZ status (SCZ = 1, Control = 0) to quantify module‐trait associations, and gene‐to‐module assignments were exported for downstream interpretation.

## 3 Results

### 3.1 Bulk Transcriptome‐Wide Expression Structure and Sample‐Level Heterogeneity

**FIGURE 1:**
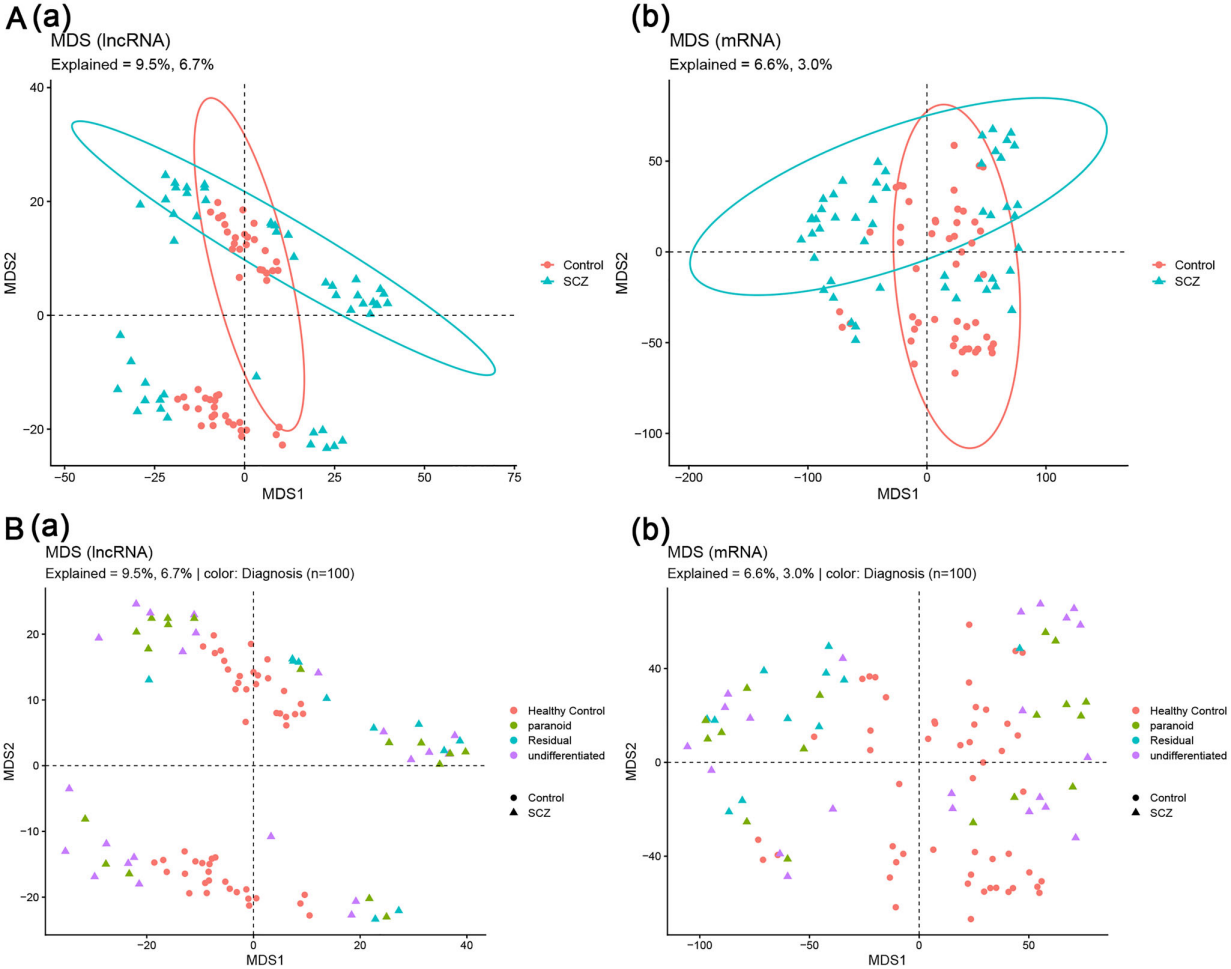
Global structure of lncRNA expression and diagnostic subtype annotation by MDS. (A) classical multidimensional scaling (MDS) based on TMMnormalized lncRNA logCPM profiles across all samples (50 schizophrenia [SCZ], 50 controls). Each point represents one individual; colors indicate diagnosis; and (B) the same MDS embedding annotated by SCZ subtypes (paranoid, undifferentiated, residual) together with controls.

Cohort demographics were well matched, with no detectable differences in age or sex distribution between SCZ cases and healthy controls (Figure S1; Supplementary Table S1). We then examined global expression structure using classical multidimensional scaling (MDS) on the TMM‐normalized lncRNA logCPM matrix. In two dimensions, SCZ and control samples partially overlapped, but SCZ samples showed visibly greater dispersion, consistent with substantial inter‐individual heterogeneity in peripheral lncRNA expression (Figure 1A). When SCZ subtypes (paranoid, undifferentiated, residual) were overlaid with controls, subtype labels did not form distinct clusters, suggesting that the observed structure is not explained by diagnostic subtyping alone (Figure 1B).

### 3.2 Differential Expression Highlights a Concise lncRNA Signature Alongside a Broader mRNA Shift in SCZ

**FIGURE 2:**
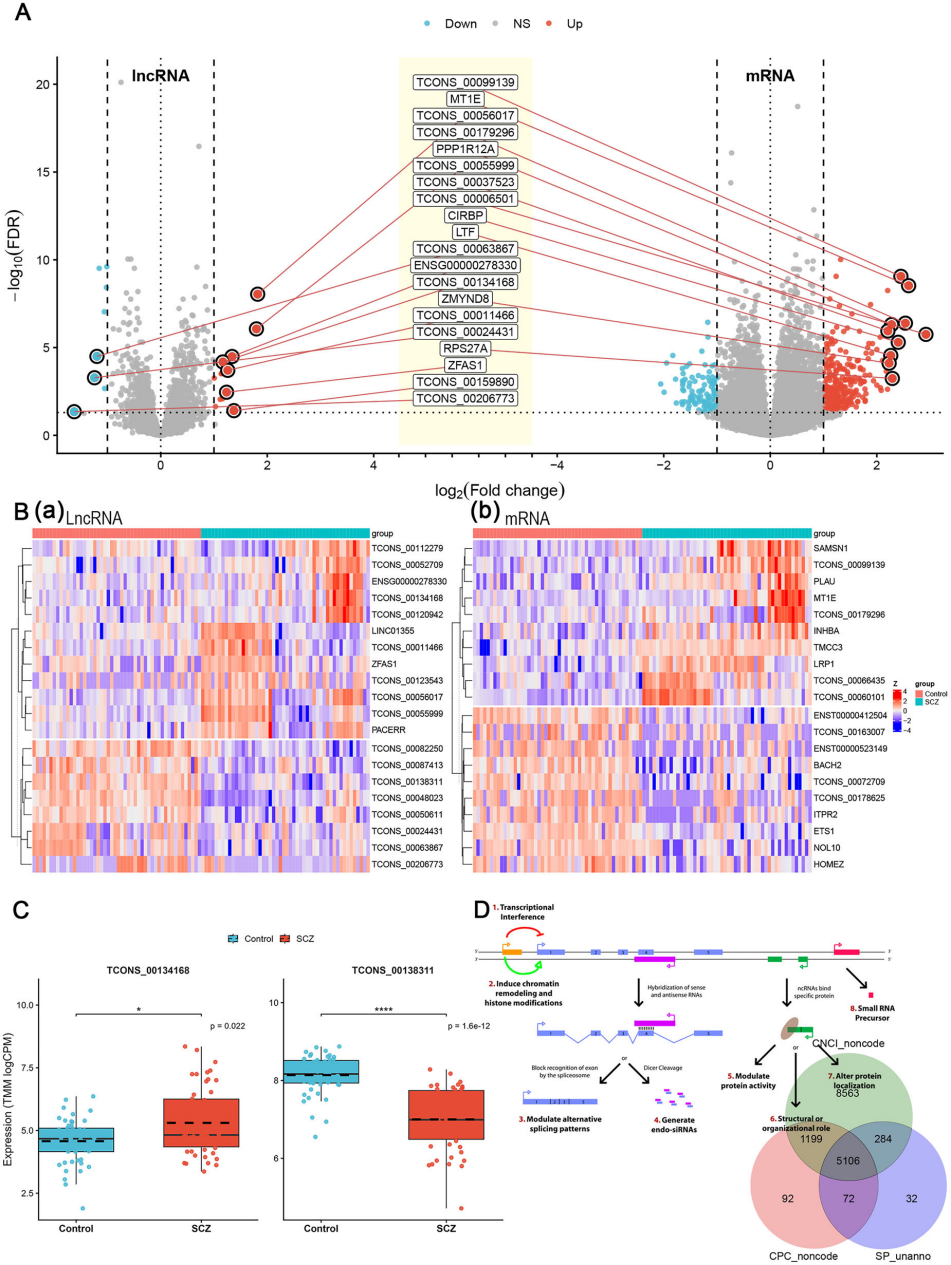
Differential‐expression overview and novel lncRNA annotation in peripheral blood leukocytes. (A) differential‐expression summary for lncRNAs and mRNAs in SCZ versus controls using edgeR (FDR < 0.05 and |log2FC| ≥ 1); (B) Heatmaps showing the top 20 differentially expressed lncRNAs and the top 20 differentially expressed mRNAs (TMM‐normalized logCPM); (C) expression of the two prioritized lncRNAs (TCONS_00134168 and TCONS_00138311/lncRNA–IL1RAP) in controls and SCZ. *P* values are shown on the plots; and (D) Summary of novel lncRNA annotation from reference‐guided transcript assembly, including the set supported as noncoding and lacking Swiss‐Prot protein annotation.

Using prespecified thresholds (FDR < 0.05 and |log2FC| ≥ 1), we identified 23 differentially expressed lncRNAs (14 upregulated, 9 downregulated) and 434 differentially expressed mRNAs (301 upregulated, 133 downregulated) in PBL from SCZ patients compared with controls (Figure 2A; Supplementary Table S7–S10). Representative high‐confidence changes from each molecular layer are shown in the MA framework (Figure S4A). Heatmaps of the top 20 dysregulated lncRNAs and the top 20 dysregulated mRNAs showed coherent group‐level shifts between SCZ and controls (Figure 2B). Two lncRNAs prioritized for downstream analyses–TCONS_00134168 and TCONS_00138311 (lncRNA–IL1RAP)–showed clear case–control differences in the discovery cohort, consistent with the genome‐wide screen (Figure 2C). In parallel, novel transcript annotation yielded a catalog of 5106 candidate novel lncRNA transcripts spanning 3716 loci, supported by noncoding evidence and the absence of protein annotation (Figure 2D).

**FIGURE 3:**
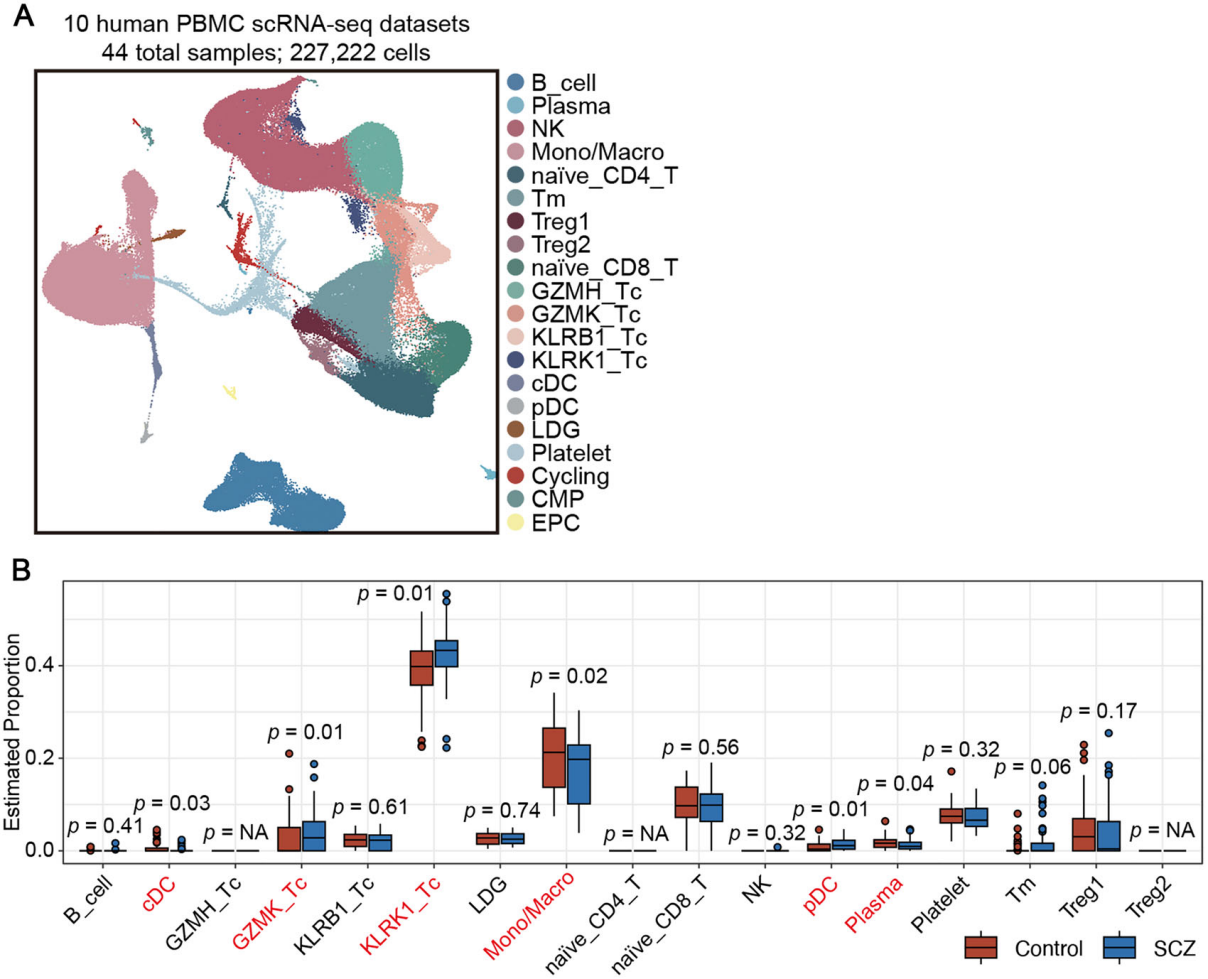
Healthy‐donor PBMC single‐cell reference atlas and bulk immune‐cell deconvolution. (A) UMAP of integrated peripheral blood mononuclear cell (PBMC) single‐cell profiles from healthy donors, used here as a reference for expression localization; and (B) estimated relative immune‐cell composition from bulk RNA‐seq by CIBERSORT, comparing SCZ and controls (only samples meeting the CIBERSORT significance criterion were retained as specified in Methods).

To add cell‐type context for the immune genes highlighted above, without invoking SCZ‐control single‐cell comparisons, we integrated public PBMC scRNA‐seq datasets from 44 healthy individuals across 10 studies to build a harmonized reference atlas (Figure 3A). Separately, we applied CIBERSORT deconvolution to bulk RNA‐seq profiles to estimate relative immune‐cell composition. This analysis suggested differences in inferred fractions—including KLRK1^+^ cytotoxic T‐cell and monocyte/macrophage‐related compartments—between SCZ and controls (*p* < 0.05; Figure 3B). Together, these results place bulk differential‐expression signals in the setting of peripheral immune‐cell heterogeneity, while keeping single‐cell data restricted to expression localization rather than disease‐state testing.

### 3.3 Expression Co‐Variation Links Prioritized lncRNAs to Reproducible Transcript‐Level Patterns

**FIGURE 4:**
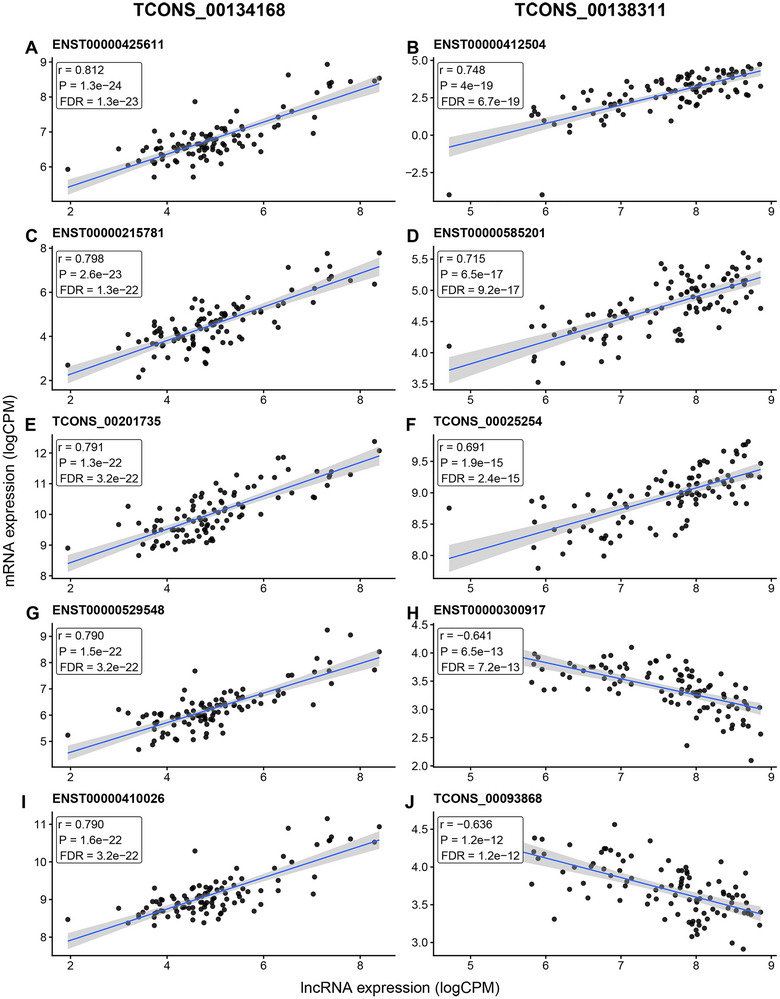
Sample‐level correlations between prioritized lncRNAs and selected mRNAs. Scatter plots show sample‐level Pearson correlations using TMM‐normalized logCPM across all individuals. TCONS_00134168 is shown in panels A, C, E, G, and I; TCONS_00138311 is shown in panels B, D, F, H, and J. Each panel reports r and the corresponding significance after multiple‐testing control as described in Methods.

To complement differential expression, we asked whether the two prioritized lncRNAs showed consistent sample‐level co‐variation with selected transcripts across individuals. TCONS_00134168 exhibited uniformly strong positive associations with five transcripts (Figure 4A,C,E,G, I; *r* = 0.790–0.812; FDR ≤ 3.2 × 10^−^^2^ ^2^). In contrast, TCONS_00138311 (lncRNA–IL1RAP) showed a broader pattern, with three positive associations (Figure 4B, D,F; *r* = 0.691–0.748; FDR ≤ 2.4 × 10^−^^1^ ^5^) and two negative associations (Figure 4H,J; *r* = −0.64; FDR ≤ 1.2 × 10^−^^1^ ^2^). We present these as correlational evidence of expression co‐variation at the individual‐sample level. They support using the two lncRNAs as compact inputs for downstream modeling, without implying direct regulatory relationships.

### 3.4 qRT–PCR Validation of Two Core lncRNAs and Reference‐Cell Mapping of the IL1B/IL1RAP/HSF1 Axis

**FIGURE 5:**
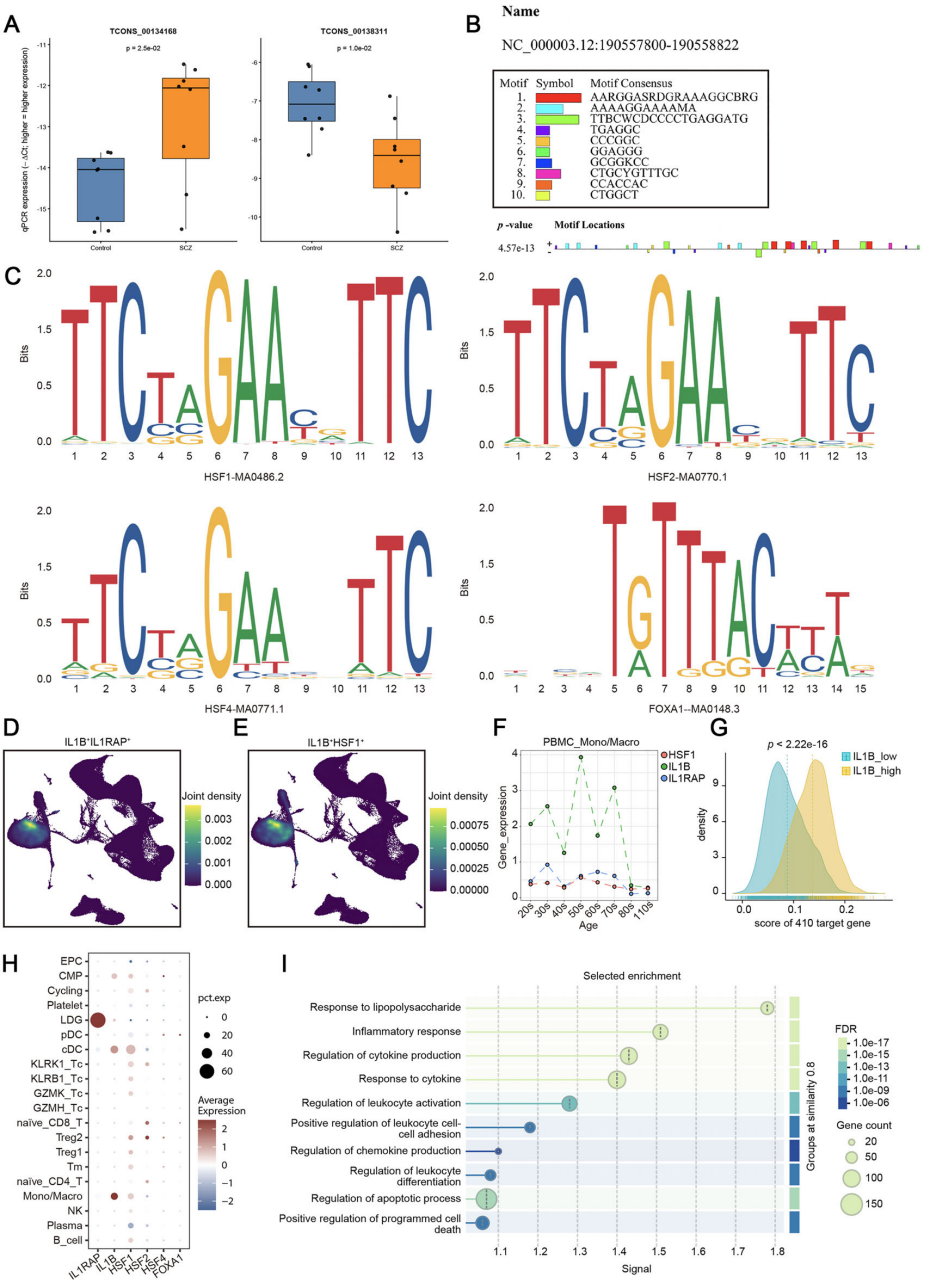
qRT–PCR validation, motif‐based annotation, and healthy‐reference single‐cell localization of the IL1B/IL1RAP/HSF1 axis. (A) qRT–PCR validation of lncRNA–CCR3 (TCONS_00134168) and lncRNA–IL1RAP (TCONS_00138311) in peripheral blood leukocytes (*n* = 16; 8 SCZ, 8 controls); β‐actin was used as the internal control and group comparisons were assessed as described in Methods; (B) genomic context of the lncRNA–IL1RAP locus and the promoter window used for motif scanning (−2000 bp to + 100 bp); (C) predicted transcription factor motifs at the lncRNA–IL1RAP promoter from JASPAR, filtered by the prespecified score criteria in methods; (D) co‐localization of IL1B and IL1RAP expression in the healthy‐reference PBMC atlas; (E) HSF1 expression in the healthy‐reference PBMC atlas; (F) descriptive age‐stratified summaries of IL1B/IL1RAP/HSF1 expression within the mono/macro compartment in healthy donors; (G) gene‐set scores of the 434 bulk differentially expressed mRNAs in IL1B^high versus IL1B^low mono/macro cells (healthy reference); (H) additional expression views of HSF1 within PBMC compartments (healthy reference); and (I) enrichment analysis of genes associated with the IL1B^high mono/macro state (healthy reference). Note: single‐cell panels are used for expression localization in healthy donors and are not SCZ‐control single‐cell comparisons.

Across the lncRNA‐mRNA co‐variation landscape, IL1B stood out as one of the most strongly associated transcripts for TCONS_00134168, while TCONS_00138311 ranked among the strongest co‐varying relationships in the lncRNA network (Supplementary Table S11). Based on genomic context and sequence similarity, TCONS_00134168 and TCONS_00138311 map to the CCR3 and IL1RAP loci, respectively; we therefore refer to them as lncRNA–CCR3 and lncRNA–IL1RAP. We then tested both candidates by qRT–PCR in PBL (*n* = 16; 8 SCZ, 8 controls). The direction matched RNA‐seq: lncRNA–IL1RAP was reduced in SCZ and lncRNA–CCR3 was increased (*p* < 0.05 for both; Figure 5A).

To add a hypothesis‐generating regulatory layer for lncRNA–IL1RAP, we screened its putative promoter region (−2000 bp upstream to +100 bp downstream) for transcription factor binding motifs. Candidates were first queried using UCSC–based annotation (Figure 5B), followed by motif scanning in JASPAR (score = 500; relative profile score threshold = 90%), retaining factors predicted in the same transcriptional direction as lncRNA–IL1RAP. This scan highlighted HSF1, HSF2, HSF4, and FOXA1 as candidate upstream regulators of the lncRNA–IL1RAP locus (Figure 5C).

To localize the signal in a cell‐type context, we used a healthy‐donor PBMC single‐cell reference atlas (for localization rather than SCZ–control single‐cell testing). IL1B and IL1RAP double‐positive cells were most frequently observed in the mono/macro compartment (Figure 5D), and HSF1 was also expressed in this compartment (Figure 5E,H). Age‐stratified summaries further showed higher IL1B/IL1RAP/HSF1 expression within mono/macro in donors in their 30 s (Figure 5F), a descriptive pattern that provides cohort context without implying disease timing. Finally, when we stratified mono/macro cells by IL1B expression, the IL1B^high stratum carried higher gene‐set scores for the 434 bulk DE mRNAs (*p* < 0.05; Figure 5G). Consistent with this mapping, genes associated with the IL1B^high state were enriched for immune‐response programs, including inflammatory response and cytokine‐related regulation (Figure 5I).

### 3.5 A Two‐lncRNA Model Yields Cross‐Validated Discrimination in RNA‐seq and Interpretable Probability Outputs in qRT–PCR

**FIGURE 6:**
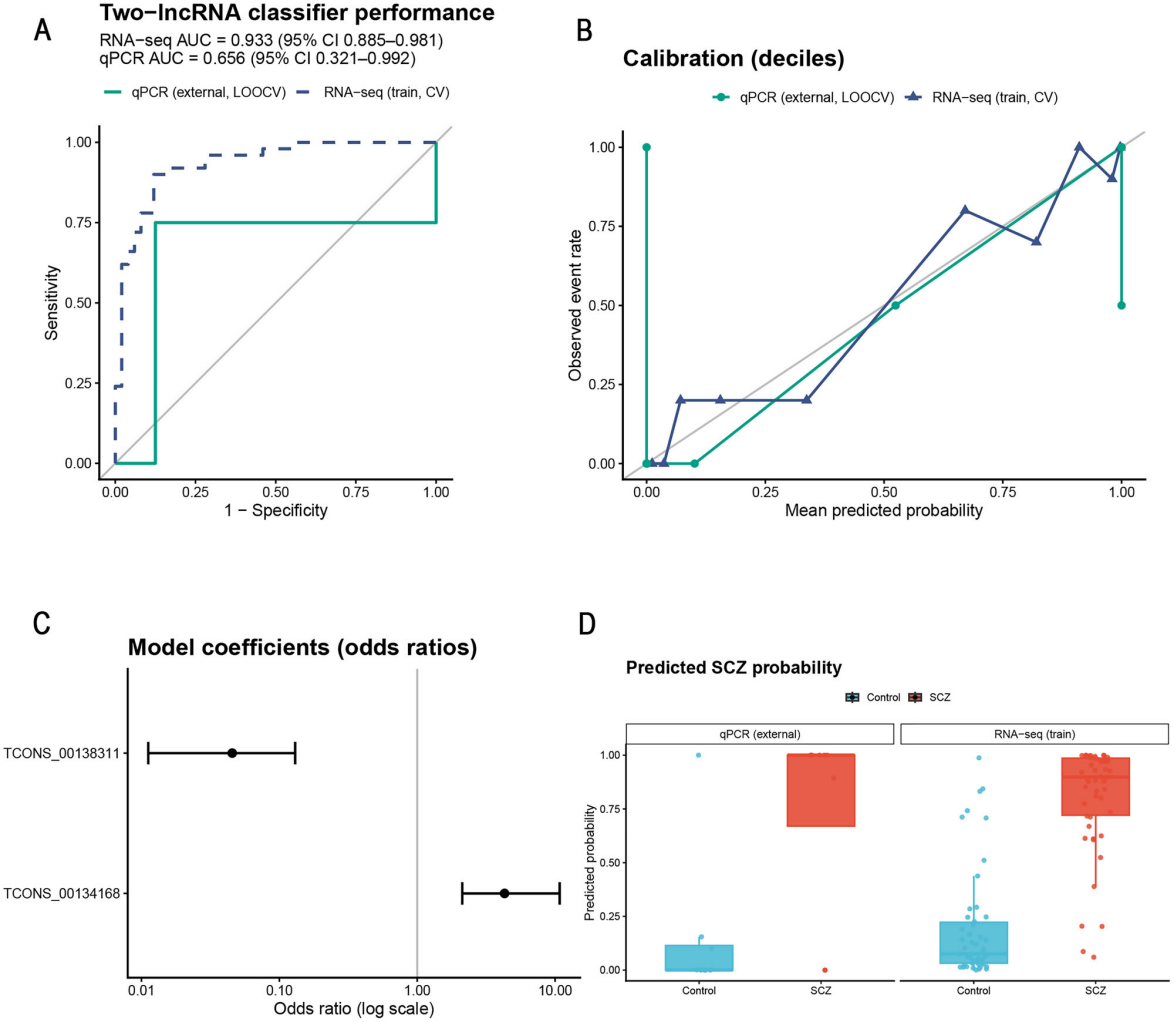
Two‐lncRNA logistic regression model and probability outputs. (A) ROC curves and AUC for the two‐lncRNA model in the RNA‐seq cohort (cross‐validation) and in the independent qRT–PCR cohort; (B) calibration curves comparing predicted probabilities with observed event rates; (C) model coefficients shown as odds ratios (ORs) with 95% confidence intervals; and (D) predicted SCZ probabilities in the qRT–RT) Predicted SCZ probabilitieseq cohort (right), shown by group (boxplots with overlaid points).

Because the two lncRNAs shifted in opposite directions between groups, we asked whether their joint signal could summarize case–control separation at the individual level. In the RNA‐seq cohort, a two‐lncRNA logistic regression model showed strong cross‐validated discrimination (AUC = 0.933, 95% CI 0.885–0.981; Figure 6A) and produced clearly separated predicted probabilities for SCZ versus controls (Figure 6D, right). The coefficient directions were consistent with these shifts: higher TCONS_00134168 corresponded to higher predicted SCZ probability (OR > 1), whereas higher TCONS_00138311 corresponded to lower predicted probability (OR < 1; Figure 6C). Resampling suggested the performance was not driven by a single favorable split (Figure S5).

When applied to the independent qRT–PCR cohort, predicted probabilities remained interpretable at the group level (Figure 6D, left), but discrimination decreased with wide uncertainty (AUC = 0.656, 95% CI 0.321–0.992; Figure 6A), consistent with a limited external sample size. Decile‐binned calibration showed partial agreement between predicted probabilities and observed event rates (Figure 6B), while the deviations—especially in the external qRT–PCR set–should be interpreted in the context of sample‐size constraints.

### 3.6 Correlation–Anchored Enrichment Analysis Reveals Opposing Translation‐Centered and IL1RAP‐Linked Immune Themes

**FIGURE 7:**
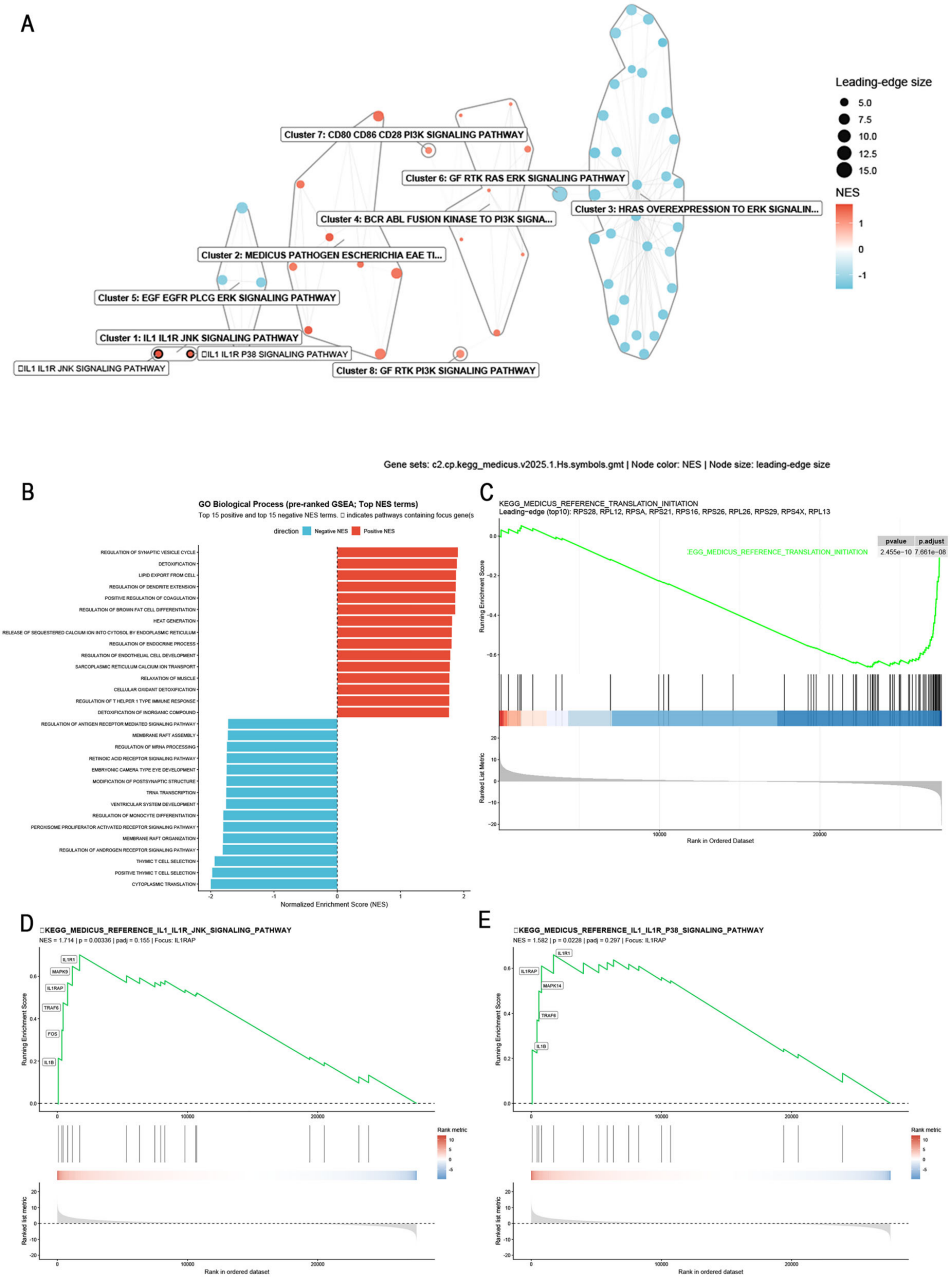
Pre‐ranked GSEA and pathway‐network summary of lncRNA‐associated mRNAs. (A) similarity network of enriched KEGG pathways based on leading‐edge overlap; pathways are grouped into modules as described in Methods, and IL1RAP‐containing IL‐1/IL‐1R branches are marked; (B) GO biological process (GO:BP) enrichment summary from pre‐ranked GSEA. (C) GSEA enrichment plot for the translation‐initiation pathway (KEGG_MEDICUS_REFERENCE_TRANSLATION_INITIATION); and (Dd nt summary from pre‐ranked GSEA. (C) GSEA enrichment plot for the translation‐initiation pathway (KEGG_MEDICUS_REFERENCE_TRANSLATION_INITIATION)taining IL‐1/IL‐1R branches are marng control follow the Methods.

We next defined a candidate gene set as mRNAs that (i) co‐varied with dysregulated lncRNAs; and (ii) were differentially expressed in the SCZ‐control comparison. Pre‐ranked GSEA of these correlation‐filtered mRNAs revealed a structured enrichment pattern within GO: BP (Figure 7B; Supplementary Table S12). Positively enriched terms emphasized synaptic‐ and neurite‐associated programs (e.g., synaptic vesicle cycling, dendrite extension) and detoxification/redox homeostasis, with NES approaching +2. In contrast, gene sets linked to core biosynthetic and RNA‐handling processes–including cytoplasmic translation and RNA processing/splicing–showed negative enrichment with NES approaching −2, alongside selected immune developmental terms. Together, these paired directions suggest that lncRNA‐associated mRNA programs in peripheral blood span both neuro‐relevant annotations and a remodeling axis centered on fundamental gene‐expression machinery.

At the pathway level, we organized enriched KEGG gene sets by constructing a similarity network based on leading‐edge overlap and grouping pathways into eight modules on a sparse backbone (Figure 7A; Supplementary Table S13). The resulting module titles captured interpretable themes (e.g., receptor tyrosine kinase–extracellular signal‐regulated kinase (RTK–ERK) signaling, PI3K‐related pathways, immune costimulatory signaling), and IL‐1/IL‐1R pathways containing IL1RAP were marked with “★”. Because edges reflect shared leading‐edge components, proximity within a module indicates shared driver genes rather than nominal pathway redundancy (Supplementary Table S14).

Among individual GSEA traces, KEGG_MEDICUS_REFERENCE_TRANSLATION_INITIATION showed strong negative enrichment (*p* = 2.455 × 10^−^^10^, FDR = 7.661 × 10^−^^8^; Figure 7C), with a leading edge dominated by ribosomal protein genes (e.g., RPS28, RPL12, RPSA, and RPS21). In parallel, two IL‐1/IL‐1R pathways containing IL1RAP showed positive enrichment, corresponding to the JNK branch (NES = 1.714, *p* = 0.00336, FDR = 0.155) and the p38 branch (NES = 1.582, *p* = 0.0228, FDR = 0.297) (Figures 7D,E). In both, IL1RAP appeared in the leading edge together with IL1B, TRAF6, IL1R1, and MAPK9/MAPK14. Although these IL‐1 pathways did not pass a strict FDR < 0.05 cutoff, their repeated appearance across network and leading‐edge views supports an IL1RAP‐linked inflammatory signaling signature in peripheral blood, which should be interpreted as suggestive evidence rather than a causal mechanism.

### 3.7 WGCNA Links lncRNA–IL1RAP and IL1RAP to SCZ‐Inversely Associated Co‐Expression Programs

**FIGURE 8:**
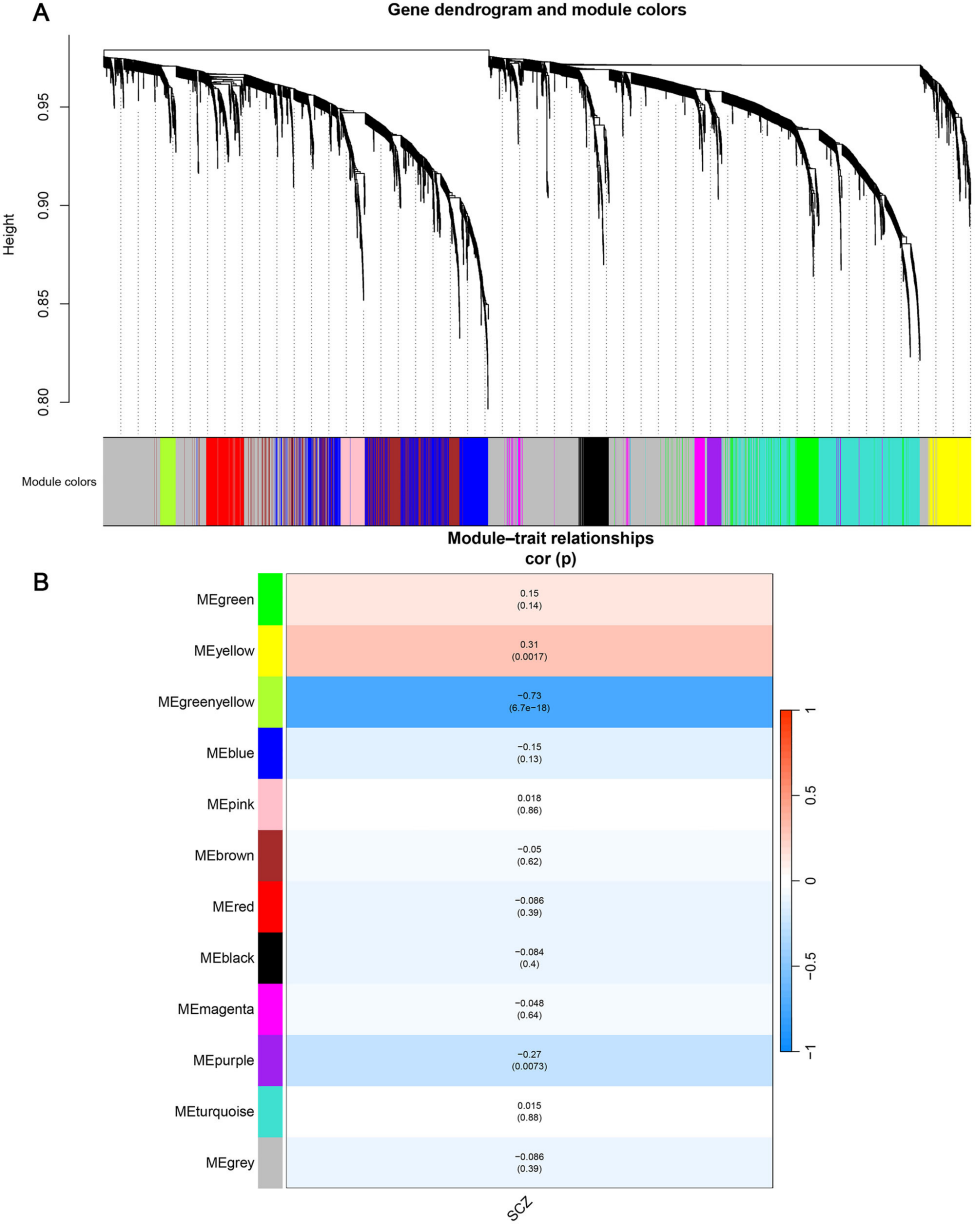
WGCNA identifies diagnosis‐associated co‐expression modules in lncRNAs and mRNAs. (A) lncRNA co‐expression network dendrogram with module assignment colors (grey indicates unassigned transcripts); and (B) module–trait relationships for lncRNA and mRNA networks, correlated with diagnosis (SCZ = 1, Control = 0); red indicates positive correlation and blue indicates negative correlation.

To move beyond single‐transcript tests, we constructed an lncRNA co‐expression network from 3862 retained lncRNA features, resolving 11 modules plus a grey set of 1421 unassigned transcripts (Figure 8A). At the eigengene level, the strongest diagnosis association was observed for the greenyellow module (*n* = 69), whose eigengene was lower in SCZ under the current coding (SCZ = 1, Control = 0; *r* = −0.73, *p* = 6.7 × 10^−^^1^ ^8^; Figure 8B). Notably, TCONS_00138311 (lncRNA–IL1RAP) was assigned to this greenyellow module, placing the candidate lncRNA within a diagnosis‐linked co‐expression program (Figures 8A–B). Two additional modules showed smaller but significant associations (yellow, *n* = 176, *r* = 0.31, *p* = 0.0017; purple, *n* = 81, *r* = −0.27, *p* = 0.0073; Figure 8B). By contrast, TCONS_00134168 localized to the pink module (*n* = 107), whose eigengene was not associated with diagnosis (*r* = 0.018, *p* = 0.86), consistent with a more transcript‐specific case–control signal under these settings (Figure 8B).

In parallel, the mRNA network grouped 8000 retained transcripts into six modules plus a large grey set. SCZ status aligned most clearly with the brown module (*r* = 0.32, *p* = 0.0012) and the turquoise module (*r* = −0.29, *p* = 0.004). IL1RAP mapped to the turquoise module, positioning it within an mRNA program whose eigengene was lower in SCZ (Figure 8B). Full module membership and gene‐to‐module assignments are provided in Supplementary Table S15–16.

## 4 Discussion

### 4.1 A Peripheral lncRNA‐Associated Transcriptome in SCZ

In this study, we combined peripheral blood leukocyte bulk RNA‐seq, a healthy‐donor peripheral blood mononuclear cell (PBMC) single‐cell reference, and sequence‐based in silico annotation to summarize lncRNA‐associated transcriptional features in SCZ. Prior work has described peripheral blood transcriptomic dysregulation in SCZ, often showing a relatively small set of lncRNA changes alongside broader mRNA remodeling, consistent with the idea that lncRNAs may shape immune and neurobiological abnormalities through regulatory networks (Wang et al. 2023). Across studies, both blood and brain RNA‐seq analyses have reported marked reconfiguration of lncRNA–mRNA co‐expression architecture, with recurrent links to oxidative phosphorylation, HIF‐1 signaling, and neurodevelopment (Long et al. 2025). Mechanistic reports further support lncRNA involvement in disease‐relevant processes–such as inflammatory signaling or synaptic cytoskeletal remodeling–without implying that any single lncRNA is universally causal (C et al. 2021; X et al. 2025). Network‐level analyses also suggest that lncRNAs can participate in competitive endogenous RNA (ceRNA) regulation that may shift immune–neural coupling at the transcriptome scale (Long et al. 2025; Teng et al. 2025). Finally, multi‐omics co‐expression and genetic association studies indicate that subsets of lncRNAs co‐localize with genome‐wide association study (GWAS) risk loci, pointing to a possible role in the molecular implementation of genetic susceptibility (Mukhopadhyay et al. 2023). Against this background, our individual‐level co‐variation analyses help place candidate lncRNAs within the broader peripheral transcriptomic perturbations observed in SCZ.

### 4.2 Two lncRNAs Form a Compact Candidate Signal

Within the overall signal landscape, we prioritized two candidate lncRNAs–TCONS_00134168 and TCONS_00138311–mapping to the CCR3 and IL1RAP genomic regions (lncRNA–CCR3 and lncRNA–IL1RAP, respectively). In case–control analyses, lncRNA–CCR3 was upregulated and lncRNA–IL1RAP was downregulated, and the direction of change was reproducible across sequencing platforms. Because these two lncRNAs move in opposite directions, joint modeling can concentrate the shared information into a compact output with clearer individual‐level interpretability. In the discovery cohort, the directionality was stable and statistically supported; in a small external validation cohort, directionality remained concordant but statistical support was weaker. Based on this pattern, the most conservative framing is a “two‐lncRNA candidate biomarker framework” that still requires confirmation in larger, multi‐center studies.

Biologically, IL1RAP and its associated lncRNA sit within an interleukin‐1 (IL‐1) signaling context that has been repeatedly discussed in neuroinflammation‐related work in SCZ. In this literature, IL‐1 signaling is linked to microglial activation and glutamate excitotoxicity, and reduced IL1RAP expression could plausibly change the balance of feedback regulation in inflammatory signaling—an interpretation that remains indirect in our dataset (Zhu et al. 2025; Pehlivan et al. 2022). Prior studies also report that IL‐1 receptor family members (e.g., IL1R2, CCR2, and CXCR4) can form heteromeric complexes with NMDA receptors and dopamine D2 receptors, providing one proposed route by which immune signaling could interface with neurotransmission (Borroto‐Escuela et al. 2017). In addition, IL1RAP acts as a shared co‐receptor across the IL‐1, IL‐33, and IL‐36 receptor complexes, linking it to multiple inflammatory signaling pathways (Grönberg et al. 2024). Genetic variation in IL1RAP has been associated with neurodevelopment–related abnormalities and NF‐κB signaling in SCZ (Cheng et al. 2022), and other reports suggest that IL1RAP–related pathways may modulate inflammatory responses, including via Th17/Treg balance (Lee et al. 2016; Cros and Segura 2025).

By contrast, non‐coding RNAs near the CCR3 locus showed an upward shift, consistent with a peripheral immune–activation pattern. CCR3 is involved in chemotactic signaling for eosinophil and monocyte recruitment, and upstream lncRNA regulation has been proposed to influence promoter activity (Fortin et al. 2010). Experimental models have linked CCR3 upregulation to neuroinflammatory features and blood–brain barrier injury in autoimmune contexts, while CCR3 reduction has been reported to mitigate neuroinflammation‐linked cognitive deficits by shifting microglial polarization and limiting immune‐cell infiltration (Zhao et al. 2024; Rege et al. 2023). Taken together, the opposite–direction changes of lncRNA–CCR3 and lncRNA–IL1RAP sketch a peripheral immune network in which chemotaxis‐related signals appear higher while IL1RAP‐linked receptor signaling appears lower; in our study, this should be read as a transcriptomic pattern that motivates stratification and testing, not as proof of mechanism or diagnostic specificity.

### 4.3 Pathway Modules Highlight RTK–ERK, PI3K, Immune Costimulation, and IL‐1 Branches

To interpret the co‐variation signal in functional terms, we analyzed enrichment among genes associated with dysregulated lncRNAs. Several processes emerged in parallel, spanning synaptic annotations, RNA processing/translation programs, and immune‐related pathways. A KEGG similarity network then grouped enriched pathways into higher‐order modules, with prominent clusters including RTK–ERK signaling, phosphoinositide 3‐kinase‐AKT (PI3K/AKT)–related pathways, and immune costimulatory signaling.

RTK–ERK and PI3K–AKT signaling are frequently discussed as core axes in neuroplasticity and cellular metabolism. In prior peripheral blood and brain studies in SCZ, altered PI3K, AKT, and CREB levels have been reported and correlated with depressive symptoms and impulsive behavior (Li et al. 2023). Animal work also suggests that PI3K/AKT/mTOR signaling can be suppressed in ketamine‐induced models and partially restored by antipsychotics, with improvements in cognitive and social phenotypes (Nawwar et al. 2022); and that activating PI3K/AKT/GSK3β signaling may reduce neuroinflammation and synaptic injury in neurodevelopmental models (Li et al. 2024). While these reports do not test our lncRNA candidates directly, they provide a plausible biological backdrop for why these modules recur in enrichment outputs.

Notably, IL‐1/IL‐1R signaling branches containing IL1RAP appeared repeatedly and aligned with JNK/p38 MAPK signaling. In prior work, IL‐1 signaling has been linked to pro‐inflammatory gene expression, microglial activation, and synaptic pruning (Zhang et al. 2022; Enache et al. 2019). Additional reports describe IL‐1β interactions with LRP1 and TLR4‐driven NF‐κB/MAPK pathways, which can amplify neuroinflammatory responses in microglia and astrocytes (He et al. 2020), and propose an endothelial cell–microglia signaling axis that promotes inflammatory cytokine expression (Zhu et al. 2019). Overall, these enrichment themes place dysregulated lncRNAs within a multi‐axis biological context, rather than pointing to a single dominant pathway.

### 4.4 Monocyte/Macrophage Compartmentalization Links Peripheral IL‐1 Signatures to Neuroimmune Inflammation

In the healthy‐donor PBMC single‐cell reference atlas, expression localization placed IL1B, IL1RAP, and HSF1 predominantly within the monocyte/macrophage (mono/macro) compartment, which provides a cell‐type context for the IL‐1‐linked features seen in bulk RNA‐seq. When mono/macro cells were stratified by IL1B expression, the IL1B‐high subset showed higher aggregate activity of the SCZ–associated gene set and aligned with annotations related to cytokine signaling, oxidative‐stress responses, and chemotactic functions. Here, single‐cell data were used for localization and functional contextualization in a healthy reference, rather than for SCZ–control differential testing at single‐cell resolution or causal inference.

Consistent with this localization, monocytes and macrophages have repeatedly been reported to exhibit a pro‐inflammatory phenotype in SCZ. Peripheral blood studies describe increased monocyte counts and an elevated monocyte‐to–high‐density lipoprotein ratio (MHR), and meta‐analyses further report higher peripheral monocyte proportion and monocyte‐to‐lymphocyte ratio (MLR) in SCZ than in controls (Muneer et al. 2023; Xue et al. 2025).

From a brain‐tissue perspective, peripherally derived, monocyte‐lineage macrophages appear to accumulate in “high‐inflammation” disease subtypes. Postmortem studies report upregulation of macrophage‐associated transcripts (e.g., CD163, CD64, FN1, and ICAM1) together with complement‐pathway molecules (C1qA, C3, and C4) in SCZ, which is compatible with a model in which peripheral monocytes access the brain and, alongside complement activation, contribute to synaptic pruning and neuroinflammation (Purves‐Tyson et al. 2020). Subtype‐focused analyses further suggest increased brain monocyte and peripheral immune‐cell markers with relatively reduced microglial markers in “high‐inflammation” SCZ subgroups, pointing to monocyte infiltration as a major component of central immune imbalance (North et al. 2021). In this setting, our single‐cell localization supports a mono/macro context for the peripheral immune signal, without implying directionality.

### 4.5 Limitations and Outlook

SCZ involves both central and peripheral biological alterations, including complement‐linked synaptic remodeling in the brain and systemic immune activation in blood, as reported across transcriptomic studies (Ruzicka et al. 2024; Emani et al. 2024; Lindholm Carlström et al. 2021; Knowles et al. 2025). Within this context, our multi‐omics framework prioritizes an IL1RAP‐linked peripheral axis as a testable neuroimmune hypothesis rather than a definitive bridge: prior work suggests that IL1RAP perturbations can influence neuronal growth and inflammatory signaling, and macrophage programs may be shaped by heat‐shock–related regulatory pathways, but directionality in SCZ requires direct functional validation (Han et al. 2024; Cheng et al. 2022; Han et al. 2024; Jego et al. 2014).

Several constraints should be emphasized. First, bulk RNA‐seq was generated from PBL, whereas single‐cell analyses used a healthy‐donor PBMC reference; therefore, cellular interpretation is limited to immune compartments shared between these sources, and granulocyte‐related inferences should be made cautiously. Second, the single‐cell layer was used for localization and contextualization in healthy individuals, not for SCZ‐control testing at single‐cell resolution. Third, biomarker performance remains exploratory: cross‐validation supports internal stability, but the external qRT–PCR cohort is small and yields wide uncertainty; larger multi‐center cohorts, longitudinal sampling, and disease‐control comparisons are needed to assess generalizability and specificity. Finally, medication history and other unmeasured exposures may still shape peripheral immune states despite washout criteria.

Looking forward, the most direct routes to strengthen inference include (i) independent, multi‐site validation with adequately powered external cohorts; (ii) matched single‐cell or single‐nucleus profiling that better reflects the leukocyte composition of bulk samples; and (iii) mechanistic experiments (e.g., CRISPRi/siRNA perturbation and ChIP‐based assays) to test whether the proposed HSF1/IL1RAP‐related regulatory features represent upstream regulation or downstream consequence in disease.

## 5 Conclusions

By integrating peripheral blood leukocyte bulk RNA‐seq with sequence‐based in silico annotation and a healthy‐donor PBMC single‐cell reference atlas for expression localization, we outline an immune‐linked, lncRNA‐associated transcriptional landscape in SCZ. Two candidate lncRNAs mapping to the CCR3 and IL1RAP loci (lncRNA–CCR3 and lncRNA–IL1RAP) show concordant case–control directionality across platforms and are supported by qRT–PCR in an independent cohort, motivating a compact two‐feature framework that yields interpretable individual‐level probability outputs. While cross‐validation supports internal stability in the RNA‐seq cohort, the attenuated and uncertain performance in a small external qPCR set indicates that any biomarker interpretation should remain exploratory pending larger, multi‐center validation. WGCNA further placed lncRNA–IL1RAP and IL1RAP within diagnosis‐inversely associated co‐expression programs, whereas lncRNA–CCR3 showed a more transcript‐specific pattern. Finally, localization of IL1B/IL1RAP/HSF1 signals to the monocyte/macrophage compartment provides a plausible cellular context for the peripheral IL‐1–linked signature, but does not establish directionality; targeted perturbation and binding assays, together with better‐powered external cohorts, are the most direct next steps to turn this convergent peripheral signature into a testable mechanism and a robust translational claim.

## Author Contributions

Zhaowei Teng, Tianhao Bao, Yong Zeng, Yunqiao Zhang conceived and supervised the project, contributed to data acquisition. Jie Wu, Ruize Niu, Zijun Liu, Yuanyuan Li designed and implemented the methods, and conducted the experiments, wrote the manuscript. All authors have approved the manuscript.

## Funding

This study was supported by the National Natural Science Foundation of China (grant numbers: 82501796), Yunnan Key Laboratory of Neuropsychiatric Disorders(202449CE340017), the Joint special fund of Applied Fundamental Research of Kunming Medical University granted by Science and Technology Office of Yunnan (CN) (grant numbers: 202101AY070001‐224), the Yunnan Education Department Fund (grant numbers: 2025J0246), and the Research Project of Yunnan Provincial Clinical Medical Center (grant numbers: 2024YNLCYXZX0280). The funding agencies had no role in the study design, data collection and analysis, decision to publish, or preparation of the manuscript.

## Ethics Statement

This study was approved by the ethics committee of the Affiliated Mental Health Center of Kunming Medical University (NO: 2019NO.01).

## Consent

Voluntary written informed consent was provided by all participants.

## Conflicts of Interest

The authors declare no conflicts of interest.

## Supporting information




**Supplementary materials**: brb371384‐sup‐0001‐SuppMat.docx

**Supplementary Tables**: brb371384‐sup‐0002‐Tables.xlsx

## Data Availability

The raw sequencing data are available from the corresponding author upon reasonable request.
