# Supplementary material for: Peripheral lncRNA–IL1RAP Dysregulation in Schizophrenia: A Multi‐Omics Bridge Between Immunity and Diagnosis
Source: Brain Behav. 2026 Apr 16;16(4):e71384. doi: 10.1002/brb3.71384 (PMC13084536; doi:10.1002/brb3.71384)
Supplement: Supplementary file 1 — Supplementary materials: brb371384‐sup‐0001‐SuppMat.docx [file BRB3-16-e71384-s001.docx]

**Supplemental material**

**1. Cohort inclusion and baseline clinical characteristics**

To assess schizophrenia-associated mRNA and lncRNA expression changes in peripheral blood leukocytes (PBL), we enrolled 50 schizophrenia (SCZ) patients and 50 healthy controls for RNA sequencing (RNA-seq). Age and sex were comparable between groups (mean ± SD age: 31.76 ± 6.11 years in SCZ vs 30.52 ± 8.04 years in controls; p = 0.38; Figure S1; Supplementary Table 1). This balance was maintained after sex stratification (Control vs SCZ: p = 0.48 in males; p = 0.77 in females) and within-group comparisons (Control: male vs female p = 0.81; SCZ: male vs female p = 0.08; Figure S1), and sex distribution did not differ between groups (Supplementary Table S1). RNA purity was checked before sequencing; OD260/OD280 values were within 1.8-2.0 (Supplementary Table S2). SCZ exclusion criteria included major comorbid medical conditions (e.g., fatty liver disease, hypertension, gout, renal failure, diabetes), alcohol/drug abuse, pregnancy or lactation, bedridden/wheelchair-bound status, and recent infection or alcohol intake within the past week. Controls were recruited under the same framework to reduce non-disease contributions to transcriptomic variation.

| 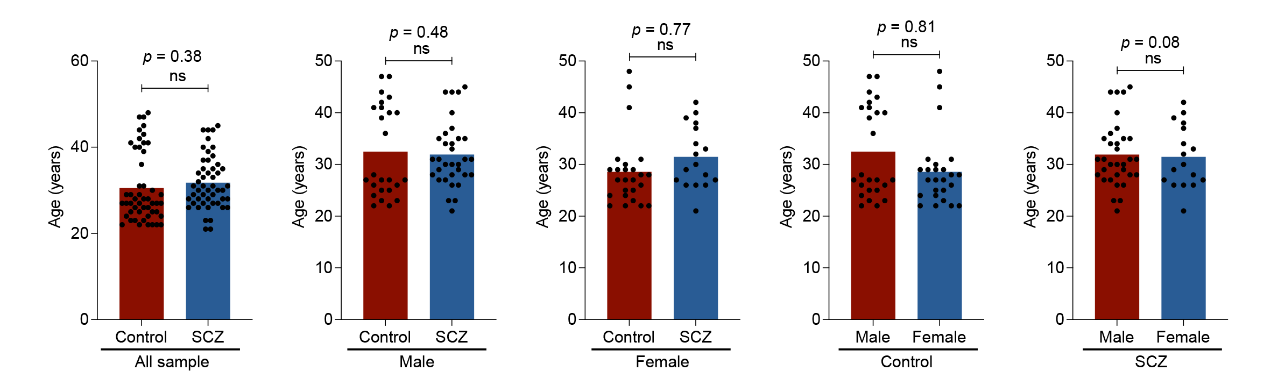 |
| --- |
| **Figure S1. Cohort matching by age and sex.**  Age and sex were balanced between schizophrenia (SCZ, n = 50) and healthy controls (n = 50). Group-level age comparison is shown with the corresponding p value (SCZ vs control, p = 0.38). Sex-stratified comparisons are also shown (male: control vs SCZ, p = 0.48; female: control vs SCZ, p = 0.77), together with within-group male–female comparisons (control: p = 0.81; SCZ: p = 0.08). Bars indicate group means where applicable. Stratified counts are provided in Supplementary Table S1. |

**2. Data filtering and sequencing quality assessment**

Clean reads were further filtered to obtain high-quality reads for downstream analyses (Supplementary Table S3). Reads were removed if they (i) contained adaptor sequences (adaptors and downstream bases were trimmed; reads shorter than 50 nt after trimming were discarded), (ii) were composed entirely of poly(A), (iii) contained >10% ambiguous bases (N), or (iv) met the low-quality criterion (≥50% of bases with Q ≤ 20). Across 100 libraries, 1.45 Tb of high-quality bases were retained from 1.48 Tb of clean bases (median retention 97.51%, IQR 97.27-97.74%), corresponding to a median of 13.34 Gb per sample (range 9.02-26.27 Gb). Base-call quality remained high after filtering (median Q20 = 97.91%; median Q30 = 93.35%, range 91.40-95.18%), with negligible ambiguous-base content (N ≈ 0%) and stable GC composition (GC 42.84-46.05%). A representative library (JS2) illustrates improved per-cycle base composition and quality after stringent filtering (Figure S2A); sequencing saturation and read-distribution diagnostics support sufficient depth and broadly uniform transcript-body coverage for this sample (Figure S2B-C).

| 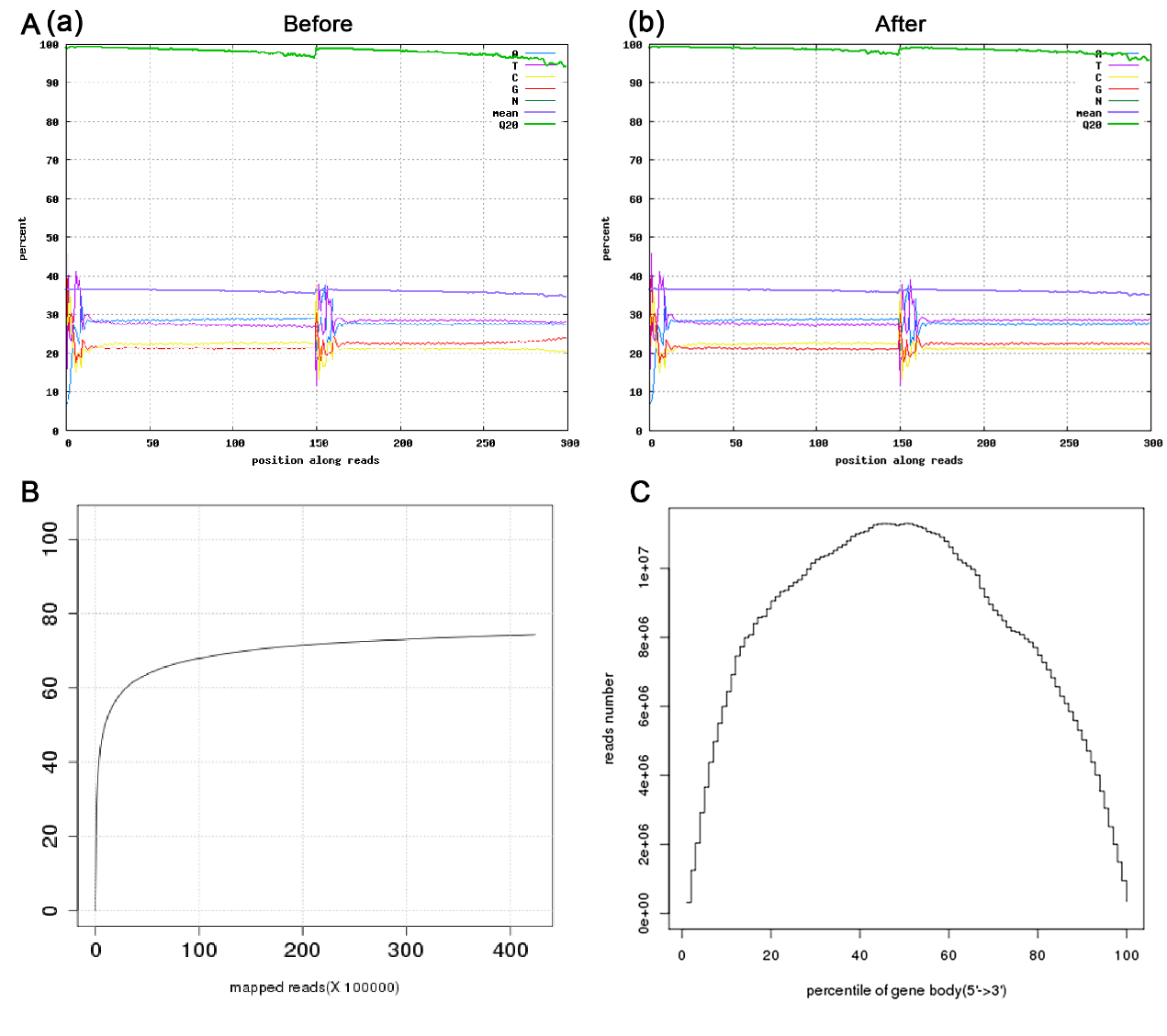 |
| --- |
| **Figure S2. Stringent read filtering and representative sequencing diagnostics (sample JS2).**  (A) Per-cycle base composition and Phred quality profiles before and after stringent filtering. Reads were removed if they contained adaptor sequence (trimmed; reads <50 nt discarded), were poly(A)-only, contained >10% ambiguous bases (N), or met the low-quality criterion (≥50% bases with Q ≤ 20).  (B) Sequencing saturation curve for JS2, showing the relationship between sequencing depth and detected transcript number; the curve approaches a plateau.  (C) Read distribution and transcript-body coverage diagnostics for JS2, consistent with largely random fragmentation and limited positional bias. Cohort-level QC summaries are provided in Supplementary Tables S2–S3. |

**3. Novel transcript identification and annotation**

To identify previously unannotated transcripts from peripheral blood leukocyte RNA-seq, reads were aligned to the human reference genome and transcript models were reconstructed in a reference-guided manner. Briefly, TopHat was used for splice-aware alignment, and Cufflinks was subsequently applied to assemble transcript structures from the alignment files. Assembled transcripts were mapped to genomic coordinates and compared to reference annotations to classify known versus novel transcript candidates. Candidate novel transcripts were retained if they met canonical long RNA criteria, including transcript length ≥ 200 nt and exon number ≥ 2, yielding a set of known transcripts and a set of newly assembled transcript models for downstream filtering and annotation.

To define a high-confidence set of novel lncRNAs, we evaluated coding potential using complementary in silico predictors, including Coding Potential Calculator (CPC) and Coding-Non-Coding Index (CNCI), and screened for protein annotation evidence against SwissProt. Novel transcript candidates were considered putative non-coding only if they were consistently predicted to lack coding potential by both CPC and CNCI and showed no protein annotation support in SwissProt. The final catalog of novel lncRNA models and their genomic annotations is provided as Supplementary Table S4, and corresponding transcript sequences are provided as Supplementary Table S5.

**4. Prediction of LncRNA Modes of Action and Expression Correlation Analysis**

Because lncRNAs are generally expressed at lower levels than mRNAs, we used correlation-based analyses to examine lncRNA–mRNA relationships across samples and visualized co-expression networks in Cytoscape32. Putative lncRNA regulatory modes were considered in three categories—antisense, cis, and trans33. For antisense prediction, RNAplex was used to identify short complementary interactions between antisense lncRNAs and mRNAs34. For cis prediction, lncRNAs located within 10 kb upstream or downstream of a gene were annotated as potential cis candidates, reflecting proximity to cis-regulatory regions. For trans prediction, we computed Pearson correlations between lncRNAs and protein-coding genes across samples and selected protein-coding genes with an absolute correlation > 0.999 (Supplementary Table S6). In addition, using DE gene symbols (|log2FC|>1, FDR<0.05) extracted from edgeR and the corresponding TMM-normalized logCPM expression matrices, we computed pairwise Spearman correlations between all DE lncRNAs and DE mRNAs across samples, and reported correlation coefficients together with two-sided p values and Benjamini–Hochberg–adjusted FDR in Supplementary Table S11.

**5. MDS annotation by demographic covariates and sequencing-depth QC**

To evaluate whether demographic variables aligned with global expression structure, we annotated classical MDS coordinates derived from the TMM-normalized lncRNA logCPM matrix by age and sex. Age did not show a clear gradient along the major MDS axes, and sex labels did not correspond to the overall sample arrangement (Figure S3A(a–b)). We observed the same pattern when repeating the analysis on the TMM-normalized mRNA logCPM matrix (Figure S3B(a–b)). Together, these annotations suggest that the dominant MDS variation more likely reflects inter-individual variability and within-group heterogeneity in peripheral transcriptomes than a single demographic covariate.

To check sequencing-depth balance and flag potential low-depth samples, we summarized library sizes from both the lncRNA and mRNA count matrices. For lncRNAs, total counts ranged from 1.03×10^6 to 2.82×10^6 (∼2.75-fold) without extreme low- or high-depth outliers, and a comparable overall distribution was observed for mRNAs. After TMM normalization, effective library sizes (library size × normalization factor) remained broadly comparable across samples with no systematic shift between SCZ and controls (Figure S3C), supporting downstream comparative analyses.

| 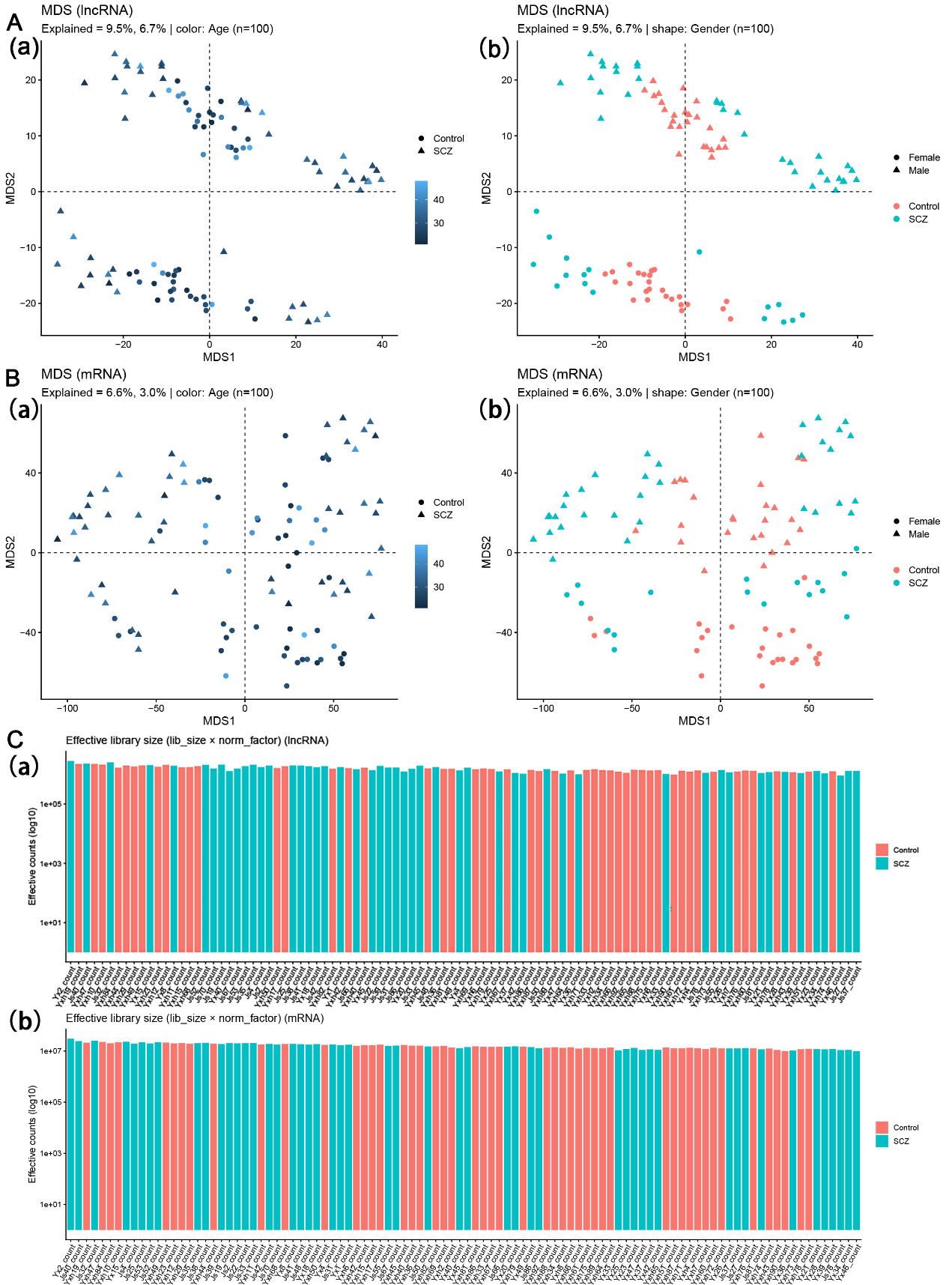 |
| --- |
| **Figure S3. Demographic annotation of global expression structure and library-size diagnostics.**  (A) Classical multidimensional scaling (MDS) computed from the TMM-normalized lncRNA logCPM matrix, annotated by (a) age and (b) sex. Percent variance explained by MDS1 and MDS2 is indicated in each panel.  (B) MDS computed from the TMM-normalized mRNA logCPM matrix, annotated by (a) age and (b) sex.  (C) Library size summaries for lncRNA and mRNA count matrices, together with TMM effective library sizes (library size × normalization factor). The x-axis indicates diagnostic group (control, SCZ), and the y-axis is shown on a log10 scale. |

**6. Differential-expression robustness across abundance and effect size**

To assess whether differential-expression (DE) calls were driven by abundance artifacts, we summarized SCZ–control contrasts using MA plots (M = log2FC; A = average expression). For both lncRNAs and mRNAs, A was defined as TMM-normalized logCPM; when logCPM/AveExpr was not available in the DE table, A was calculated as the across-sample mean of TMM logCPM from the original count matrix. Under the predefined criteria (FDR < 0.05 and |log2FC| ≥ 1), significant features separated by direction in MA space (Figure S4A). The two highlighted lncRNAs were detected at appreciable abundance and followed their expected directions: TCONS_00138311 localized to negative M (log2FC = −1.01, FDR = 2.47×10⁻¹⁰), whereas TCONS_00134168 localized to positive M (log2FC = 1.16, FDR = 3.24×10⁻⁴), arguing against low-abundance noise as the primary explanation.

We also plotted the same DE results in volcano space to view effect size alongside statistical support and to contextualize prioritized genes relative to the genome-wide distribution (Figure S4B). Among mRNAs, IL1B showed a robust increase (log2FC = 1.45, FDR = 7.69×10⁻⁷). We additionally highlighted IL1RAP, HSF1, REL, and BCL3 for side-by-side comparison within the same framework. Boxplots of TMM logCPM further illustrate sample-level directionality (Figure S4C), with clear IL1B and BCL3 upregulation and an increase in REL, whereas IL1RAP and HSF1 showed comparatively modest between-group shifts in this cohort.

| 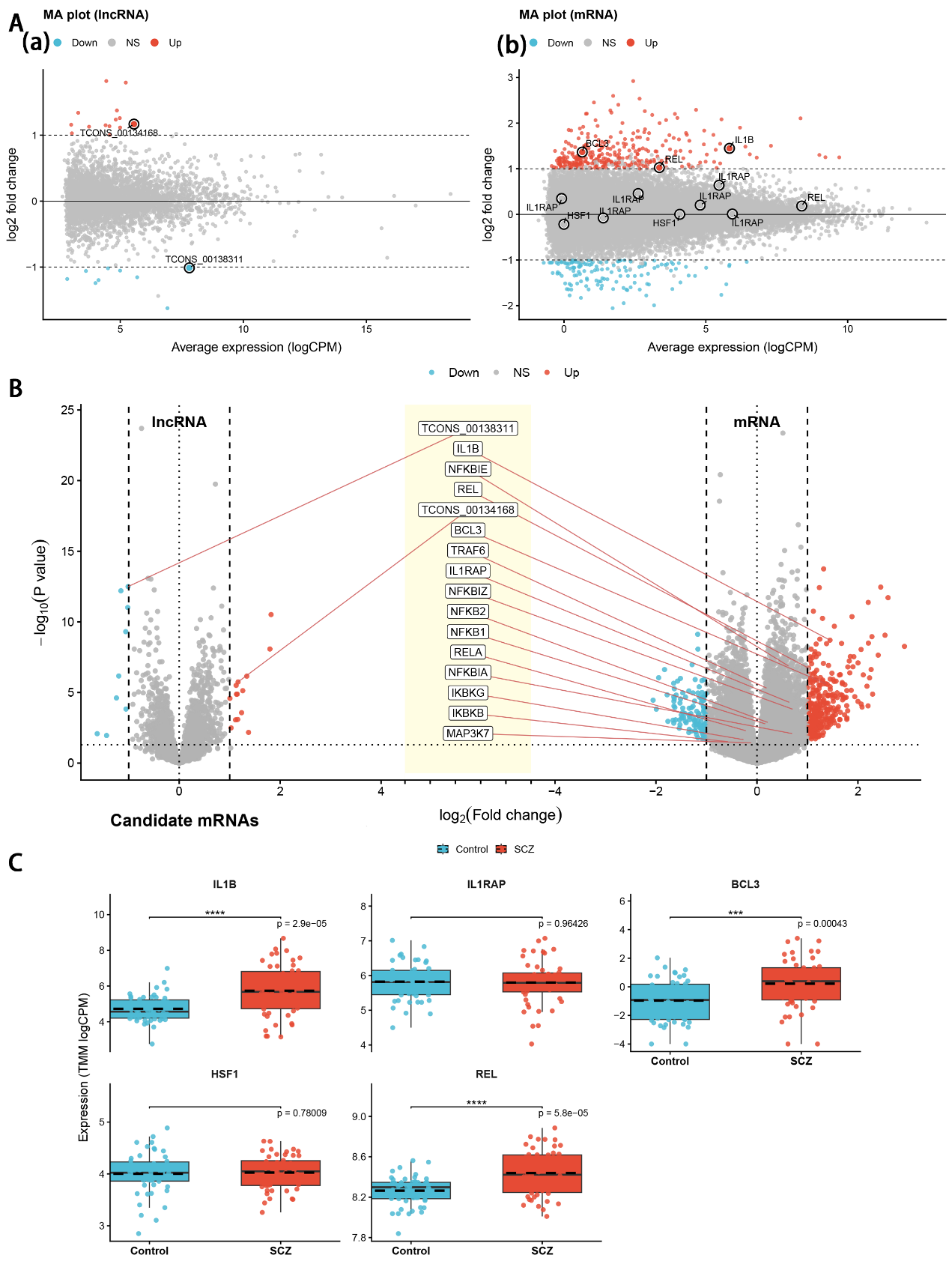 |
| --- |
| Figure S4. Differential-expression patterns across abundance and effect size.  (A) MA plot for SCZ versus controls (M = log2FC; A = average expression), where A denotes TMM-normalized logCPM (or the across-sample mean of TMM logCPM if logCPM/AveExpr was not present in the exported table). Differential expression was defined as FDR < 0.05 and \|log2FC\| ≥ 1.  (B) Volcano plot showing effect size and statistical support; selected genes are highlighted for context (including IL1B, IL1RAP, HSF1, REL, and BCL3).  (C) Boxplots of TMM-normalized logCPM for selected genes across diagnostic groups. Boxes show the interquartile range with the median line; p values are shown as annotated. |

**7. Resampling stability of the two-lncRNA diagnostic model**

Within the RNA-seq training cohort, we assessed resampling stability of the two-lncRNA logistic regression model using 50 repeats of 10-fold cross-validation. AUC values clustered within a narrow range (Figure S5), suggesting that model discrimination was not driven by a single favorable split.

| 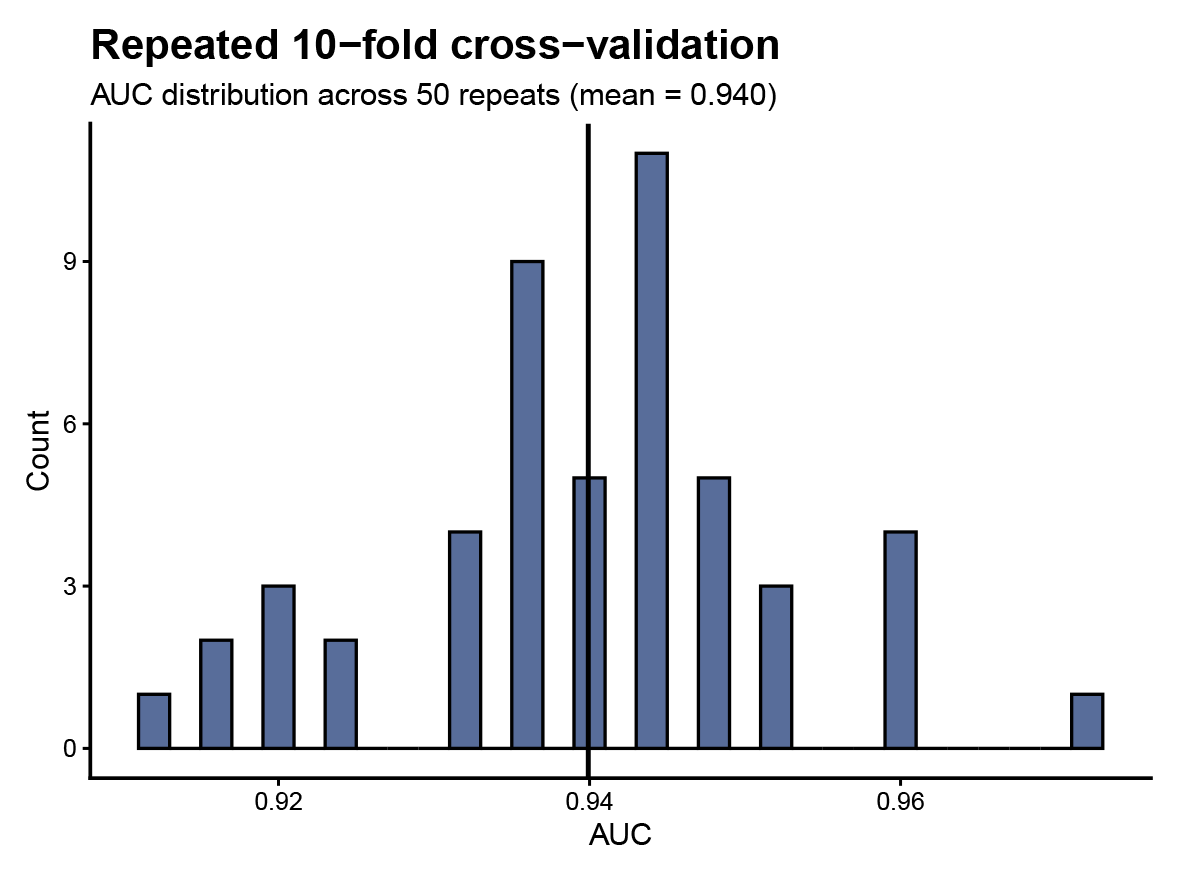 |
| --- |
| **Figure S5. Resampling stability of the two-lncRNA logistic regression model.**  Distribution of AUC values from 50 repeats of 10-fold cross-validation in the RNA-seq training cohort; the vertical line indicates the mean AUC (annotated). |

**8. WGCNA quality diagnostics support robust network construction**

Before constructing the lncRNA co-expression network, we examined sample-level clustering and soft-threshold diagnostics to reduce sensitivity to atypical samples and to choose a parsimonious power for a signed network. Hierarchical clustering did not identify an extreme outlier profile, supporting inclusion of all individuals in WGCNA (Figure S6A). Across candidate powers, the signed scale-free topology fit increased toward the target line (signed R²≈0.85) at β=6 while mean connectivity remained non-trivial; higher powers provided limited additional gain in fit but further reduced connectivity (Figure S6B). These diagnostics support stable module detection under the selected parameters.

| 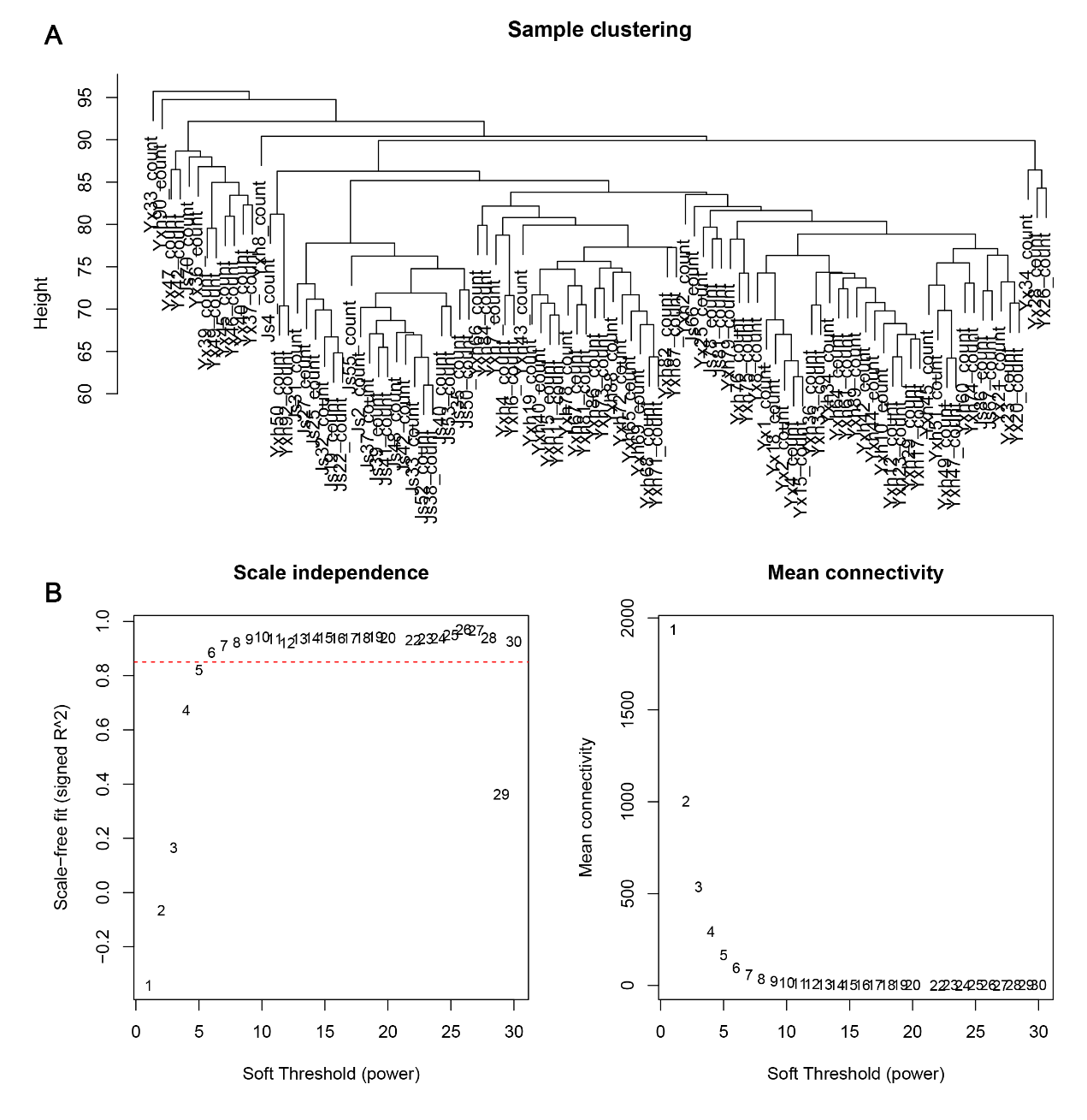 |
| --- |
| **Figure S6 \| WGCNA quality checks for lncRNA network construction.**  (A) Sample clustering used to screen for extreme outliers prior to network construction.  (B) Soft-threshold diagnostics for selecting the signed network power (β), showing scale-free topology fit (signed R²) and mean connectivity as a function of power. The selected power (β = 6) balances scale-free fit and connectivity for downstream module detection. |
